# Community Structure, Drivers, and Potential Functions of Different Lifestyle Viruses in Chaohu Lake

**DOI:** 10.3390/v16040590

**Published:** 2024-04-11

**Authors:** Yu Zheng, Zihao Gao, Shuai Wu, Aidong Ruan

**Affiliations:** 1 The National Key Laboratory of Water Disaster Prevention, Hohai University, Nanjing 210024, China; 211301010120@hhu.edu.cn (Y.Z.); 211301020001@hhu.edu.cn (Z.G.); swu_hhu@hhu.edu.cn (S.W.); 2 College of Hydrology and Water Resources, Hohai University, Nanjing 210098, China; 3 College of Geography and Remote Sensing, Hohai University, Nanjing 210098, China

**Keywords:** Chaohu Lake viruses, virus community structure, lytic viruses, temperate viruses, virus–host linkage, auxiliary metabolic genes (AMGs)

## Abstract

Viruses, as the most prolific entities on Earth, constitute significant ecological groups within freshwater lakes, exerting pivotal ecological roles. In this study, we selected Chaohu Lake, a representative eutrophic freshwater lake in China, as our research site to explore the community distribution, driving mechanisms, and potential ecological functions of diverse viral communities, the intricate virus–host interaction systems, and the overarching influence of viruses on global biogeochemical cycling.

## 1. Introduction

Viruses, estimated at around 10^30^ species globally, are the most prevalent organisms on Earth [1,2]. Viruses play a key regulatory role, affecting the stability and function of ecosystems. This role is important for maintaining ecological equilibrium and mitigating host overpopulation.

Recent advancements in aquatic ecosystem virus ecology have surged remarkably. Notably, significant strides have been made in marine environments [3,4,5], estuaries [6,7], and the epilimnion of lakes [8,9,10,11]. Microbes in sediment, a crucial component of aquatic ecosystems, exhibit extraordinarily high densities [12] and significantly influence the biogeochemical cycling of elements, thereby playing a pivotal role in maintaining the Earth’s ecosystem equilibrium [13]. However, our understanding of virus distribution, the driving factors behind their prevalence, and their potential roles in sediment remains constrained by the heterogeneity of soil and its compositional complexity, among other factors. Recent investigations into sedimentary virus communities have revealed that in estuarine and proximal coastal zones, variations in virus communities are markedly associated with environmental parameters such as pH and salinity [6]. Depth and seasonal variations are identified as key determinants of virus community structure diversity in Lake Baikal [9]. In addition, environmental microorganisms, including bacteria and archaea, constitute the foundational basis for virus sustenance and multiplication, significantly impacting on virus distribution. Che [8] has shown that nutrient levels and prokaryotic community structures serve as critical drivers for the diversity in viral community structures within freshwater lake waters. However, explorations into freshwater lake sediments remain scarce. In addition, the majority of exiting studies aggregate viruses into a whole community, seldom delving into the distinct distribution patterns of viruses and the influences shaping their lifestyles.

Viruses, based on their lifecycle, can be classified into lytic and temperate categories, each infesting distinct hosts and exerting divergent ecological effects. Lytic viruses, upon infecting a host, initiate rapid replication, culminating in host cell lysis and the subsequent release of organic matter, thereby nourishing surrounding biota [14]. In contrast, temperate viruses, under certain environmental stimuli, may integrate their genomic material into the host’s chromosome or achieve a stable intracellular symbiosis [15]. Notably, viruses can significantly alter host ecological functions through the auxiliary metabolic genes (AMGs) they carry. AMGs were initially identified in the completely sequenced genomes of cultured phages infected with marine heterotrophs [16] and cyanobacteria [17]. Recent studies in deep sea [18], freshwater [10], wetland [19], and mangrove soils [20] have found that viruses carry a large number of AMGs related to carbon, nitrogen, and sulfur cycling, which may indirectly or directly affect biogeochemical cycles. Some temperate viruses can enhance the host’s adaptability and resilience in adverse environmental conditions [21,22,23], and augment bacterial virulence through the expression of AMGs. For example, the diphtheria toxin of Corynebacterium diphtheriae is essentially an expression product of the tox gene of the phage [24]. Viruses with different lifestyles in the ecosystem may carry different AMGs, but the current understanding of this remains limited. A better understanding of the community structure and differences in AMGs carried by lytic and temperate viruses could reveal their microbial and ecological impacts in freshwater lakes.

In this research, Chaohu Lake, one of largest freshwater lakes in China, served as the focal point of our study. We selected two regions within the lake, characterized by distinct trophic conditions in its eastern and western centers, as our primary sampling sites. Samples of overlying water and sediment were collected in situ for 16S rRNA and metagenomic sequencing analyses. The aims of this study were as follows: (1) investigate the distribution, composition, and diversity of virus communities with different lifestyles in Chaohu Lake; (2) explore the influence of biotic and abiotic factors on these diverse virus communities; and (3) identify the host differences among viruses of varied lifestyles in Chaohu Lake, including the auxiliary metabolic genes (AMGs) they harbor. This investigation marks the inaugural comprehensive analysis of virus communities of differing lifestyles in Chaohu Lake through a metagenomic lens, aiming to unveil the intricate dynamics of virus–host interactions and metabolic pathways. The insights gleaned from this study promise to enrich our understanding of viral ecology in freshwater lakes, offering a novel perspective on the role viruses play in such ecosystems.

## 2. Materials and Methods

### 2.1. Sample Collection and Processing

We established two sampling and monitoring zones in Chaohu Lake, i.e., the eastern lake center zone and the western lake center zone (CES: 31°33′13″ N, 117°39′7″ E; CWS: 31°37′51″ N, 117°22′46″ E, Figure S1). Further details are provided in the Supplementary Materials. Within each sampling and observation area, three parallel sampling sites were designated, spaced 3–5 m apart to ensure spatial variability. The chosen sites are characterized by stable environmental conditions, significantly removed from near-shore hydraulic disturbances, ensuring the integrity of the samples. Lake water samples were collected from each site at depths of 1 m, 2 m, 3 m, and 4 m below the water surface in November 2021. These samples were subsequently combined in sterile bags for homogenization. Approximately 45 cm of columnar substrate was collected in situ vertically, stratified every 5 cm. Each stratified section was then bagged and mixed to ensure representativeness. Upon collection, each sample was promptly transported to the laboratory under controlled low-temperature conditions. Within a sterile environment, each sample was divided into two equal aliquots for storage at −80 °C and 4 °C, pending DNA extraction and physicochemical analysis. Considering the similar microbial community composition of the replicate samples at each site, three replicates from the same site were mixed into one sample for 16S rRNA and metagenomic sequencing. This approach yielded 18 lake sediment samples and 2 lake water samples for comprehensive examination.

### 2.2. Sample Physicochemical Measurement

Physicochemical indicators such as temperature, pH, turbidity, conductivity moisture content, and salinity were determined in situ. Total nitrogen (TN), ammonia nitrogen (NH_4_^+^-N), nitrate nitrogen (NO_3_^−^-N), nitrite nitrogen (NO_2_^−^-N), and total phosphorus (TP) were determined as described previously [25,26]. Dissolved organic carbon (DOC) was determined using a total organic carbon analyzer (TOC-L CPH, Daojin, Suzhou, China) (Table S3).

### 2.3. DNA Extraction, Sequencing, and Metagenome Assembly

As described previously [25], we performed 16S rRNA and metagenomic sequencing on 2 water samples and 18 sediment samples (0–45 cm). The V4–V5 region was amplified using the 515F (5′-GTGYCAGCMGCCGCGGTAA-3′) and 926R (5′-CCGYCAATTYMTTTRAGTTT-3′) primers. Each sample was assembled separately using MAGAHITv1.1.3 [27] default parameters. Assembly quality was assessed using QUAST v5.0.2 [28] (Table S2).

The contigs in each sample were grouped using the binning module of metaWRAP v1.3.2 [29]. Subsequently, these contigs were merged into a final bin set employing the Bin_refinement module with parameters set to -c 50 and -x 10. All resulting bin sets underwent aggregation and de-replication at 95% average nucleotide identity (ANI) using dRep v3.2.3 [30]. This process yielded 58 species-level metagenome-assembled genomes (MAGs). To classify each MAG, GTDB-tk v2.1.1 [31] was employed in conjunction with the Genome Taxonomy Database R207_v2.

### 2.4. Identification of Viral Contigs

Viral sequences were extracted from metagenome assemblies using VirSorter v2.2.4 [32] and DeepVirFinder v1.0 [33]. Contigs meeting specific criteria were selected for further analysis: they had to possess a VirSorter score >0.9 and DeepVirFinder score ≥ 0.9 and a minimum length of 10 kb. Subsequently, these contigs were grouped into viral operational taxonomic units (vOTUs) based on a 95% nucleotide identity threshold using CD-HIT v4.6.8 [34] with parameters set as follows: -c 0.95 and -aS 0.85. The integrity of the viral genome was evaluated using the CheckV v1.0.1 pipeline [35]. Furthermore, temperate viruses were identified through the presence of lysogeny-specific genes or prophage signals within viral contigs, utilizing both CheckV and VIBRANT v1.2.1 [36]. Other vOTU lifestyles are presumed to represent potential lytic viruses. The bioinformatic process is represented using a flow chart (Figure S2).

### 2.5. Taxonomy Assignment

Chaohu vOTUs was classified using PhaGCN2.1 [37] with default parameters. Open reading frames (ORFs) from vOTUs predicted by Prodigal v2.6.3 [38] were compared against the NCBI Viral RefSeq database utilizing BLASTp (e-value < 0.0001, bit score ≥ 50). Subsequently, following the methodology described previously [39,40], the BLASTp v2.6.0 outputs were imported into MEGAN v6.24.22 [41] for classification analyses employing both a majority rule approach and a lowest common ancestor algorithm. In cases of conflicts, the results of the majority rule method were given precedence after merging the outcomes of both classification methods.

Gene-sharing networks were constructed using vConTACT2 [42]. The dataset includes NCBI Viral RefSeq version 85, viral contigs from cold seeps [40], wetland [19], and permafrost [43]. This approach allows sequences to be assigned to viral clusters (VCs) at the genus level. For visualization, we employed Cytoscape v3.9.1 [44], utilizing an edge-weighted spring embedding model to represent the network.

### 2.6. Abundance of Viruses

Reads per kilobase per million mapped reads (RPKM) values were used to represent the relative abundance of viruses. Using BamM v1.7.3 ‘make’ and ‘filter’ modules, we processed the data with the parameters set to --percentage_id 0.95 --percentage_aln 0.75. The resultant file was used to generate the relative abundance of each sample using CoverM v0.3.2 (https://github.com/wwood/CoverM) accessed on 20 May 2023, with the parameters set as follows: --trim-min 0.10 --trim-max 0.90 --min-read-percent-identity 0.95 --min-read-aligned-percent 0.75 -m rpkm.

### 2.7. Virus–Host Prediction

Using iphop v1.3.2 [45], in-situ-assembled MAGs (*n* = 58) were added and constructed using three publicly available genome sets set by the software defaults, i.e., the GTDB database (release 201) [46], genomes published in the IMG database (as of 7 July 2021) [47], and genomes from Earth’s Microbiomes (GEM) [48] to maximize the prediction of potential hosts for the virus. We used ‘Blast’, ‘CRISPR’, ‘WIsH’, ‘VirHostMatcher’, and ‘PHP’ methods to obtain results with high confidence.

### 2.8. Identification of Auxiliary Metabolic Genes

We initially employed VirSorter2 (--prep-for-dramv) to identify viral sequences, followed by annotation with the default DRAM-v v1.4.6 database [49]. Within the DRAM-v output, only putative AMGs scoring < 4 were retained. AMG predictions were generated using the default parameters of VIBRANT. Subsequently, manual screening was conducted to enhance accuracy, focusing primarily on eliminating nucleotide metabolism enzymes, glycoside hydrolases, peptidases, glycosyltransferases, adenylyltransferases, transferase methyltransferases, and ribosomal proteins [50]. Following previous definitions [51], all identified AMGs were categorized into two groups: class I AMGs were genes involved in ‘metabolic pathways’ as defined in the Kyoto Encyclopedia of Genes and Genomes (KEGG), while genes belonging to other pathways were considered as class II AMGs. To mitigate the influence of host reads on the abundance estimation of virus-encoded AMGs, we characterized AMG abundances based on vOTU abundances.

### 2.9. Statistical Analyses

All statistical analyses were performed in R v4.2.2. Alpha and beta diversity of virus communities were calculated using the vegan package. Student’s *t* test was used to test the significance of differences in the structure of virus communities at different depths in Chaohu Lake. Beta diversity was calculated using Bray–Curtis for vOTU table distances, and significance was tested by analysis of variance (PERMANOVA). Mantel’s test was used to compute the different lifestyle viral communities’ correlation with environmental factors. Graphs were plotted using Prism v9.5.0 and the online platform (https://www.chiplot.online/) accessed on 10 March 2024.

## 3. Results

### 3.1. Community Structure of Prokaryotic Microorganisms in Chaohu Lake

**Figure 1:**
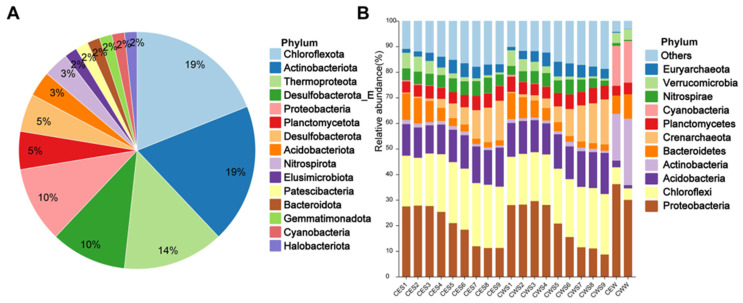
Microbial community structure in Chaohu Lake. (**A**) The numbers of MAGs classification. (**B**) Community compositions (phylum level) of bacterial and archaea by 16S rRNA. The groups are labeled as follow: ‘CE’ and ‘CW’ means the central part of the eastern and western Chaohu Lake, respectively. The final letters ‘S’ and ‘W’ stand for sediment and lake water, respectively. Numbers from small to large represent layers from surface to deeper water.

The two lake water samples and eighteen sediment samples previously described underwent metagenomic sequencing, each generating approximately 6.5 Gbp of clean data. The header assembly of these metagenomic sequencing data yielded 58 high- or middle-quality MAGs with ≥50% integrity and ≤10% contamination. These MAGs, with a 95% average nucleotide identity (ANI) clustering, represent 15 phyla, including 49 bacterial and 9 archaeal MAGs (Figure 1A; Table S4). Most bacterial MAGs belonged to *Actinobacteriota* (*n* = 11), *Chloroflexota* (*n* = 11), *Desulfobacterota_E* (*n* = 6), and *Proteobacteria* (*n* = 6). Archaeal MAGs belonged to *Thermoproteota* (*n* = 8) and *Halobacteriota* (*n* = 1).

Subsequent 16S rRNA sequencing of the same samples revealed that *Proteobacteria* (8.5–29.6%), *Chloroflexota* (18.6–24.7%), and *Acidobacteriota* (10.8%-15.9%) were the three phyla demonstrating the highest bacterial abundance in the sediments of Chaohu Lake. Conversely, in the lake water, *Proteobacteria* (27.1–41.5%), *Actinobacteriota* (14.9–29.0%), and *Cyanobacteria* (12.0–21.9%) emerged as the most abundant bacterial phylum (Figure 1B). Regarding archaea, *Crenarchaeota* (0.4–17.6%) and *Euryarchaeota* (1.3–5.8%) were identified as the predominant archaeal phyla in sediments, unlike their negligible relative abundance in the lake water. Comparative analysis did not reveal any significant differences in community composition between the eastern and western regions of Chaohu Lake at identical sediment depths (Figure 1B). The congruence between the findings from MAGs and 16S rRNA sequencing through metagenomic assembly substantiates the data and lays a solid foundation for further exploration of the interactions between viruses and their prokaryotic hosts in Chaohu Lake.

### 3.2. Community Composition of Viruses in Chaohu Lake

The above 20 metagenomic reads were assembled into contigs, which yielded 9192 putative viral sequences. After VirSorter and Deepvirfinder screening, we obtained 132 viral sequences from the sediment samples and 665 viral sequences from the lake water samples, and finally obtained 670 vOTUs (Table S5), suggesting that there were different viral sequences in sediment and lake water. This phenomenon is consistent with previous studies [5]. The vOTUs completeness was assessed using CheckV, yielding 11 (1.6%) complete, 15 (2.2%) high-quality, 62 (9.3%) medium-quality, and 559 (83.4%) low-quality samples (Table S5; Figure S3).

**Figure 2:**
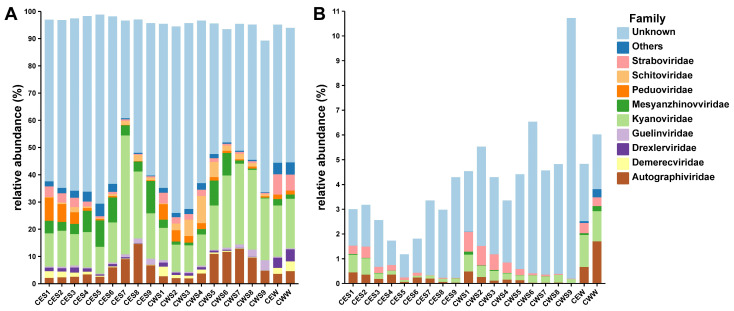
Viral community structures. Community compositions (family level) of lytic (**A**) and temperate (**B**) viruses. The groups are labeled as follow: ‘CE’ and ‘CW’ means the central part of the eastern and western Chaohu Lake, respectively. The final letters ‘S’ and ‘W’ stand for sediment and lake water, respectively. Numbers from small to large represent layers from surface to deeper water.

We performed species identification of 670 vOTUs obtained from the Chaohu Lake ecosystem by PhaGCN2 and common ancestry methods, and classified 310 vOTUs, accounting for 46.3% of the total vOTUs, of which *Kyanoviridae* (*n* = 123), *Straboviridae* (*n* = 48), and *Autographiviridae* (*n* = 35) were the most prevalent. A total of 23 vOTUs were obtained by CheckV and VIBRANT as temperate viruses, while the remaining 647 vOTUs were inferred to be lytic viruses. As anticipated, the cumulative relative prevalence of lytic viruses (comprising 89%–98% of the overall virus population) exhibited a notable elevation compared to temperate viruses. There was a higher diversity of lytic virus species. The viral communities of both types were primarily governed by the order *Caudovirales*, albeit displaying distinct community compositions (Figure 2A,B; Table S6). *Kyanoviridae* exhibited the highest abundance among both temperate viruses and lytic viruses. In the case of lytic viruses, *Autographiviridae* predominated in the sediments, while *Straboviridae* showed higher abundance than *Autographiviridae* in the lake water. Regarding temperate viruses, the relative abundances of *Kyanoviridae* and *Straboviridae* decreased with sediment depth, and the relative abundances of *Kyanoviridae* and *Autographiviridae* were higher in lake water than sediments. The majority of temperate viruses (89.1%) and lytic viruses (92.7%) were found in both sediment and lake water, with shared vOTUs gradually decreasing with depth. A total of 600 (89.5%) vOTUs were present in both the eastern and western Chaohu Lake, suggesting a high degree of similarity due to lake connectivity (Figure S4). However, variations in contamination levels, hydrological conditions, and other specificities may contribute to differences between the eastern and western central areas of the lake.

### 3.3. Diversity of Chaohu Viruses

**Figure 3:**
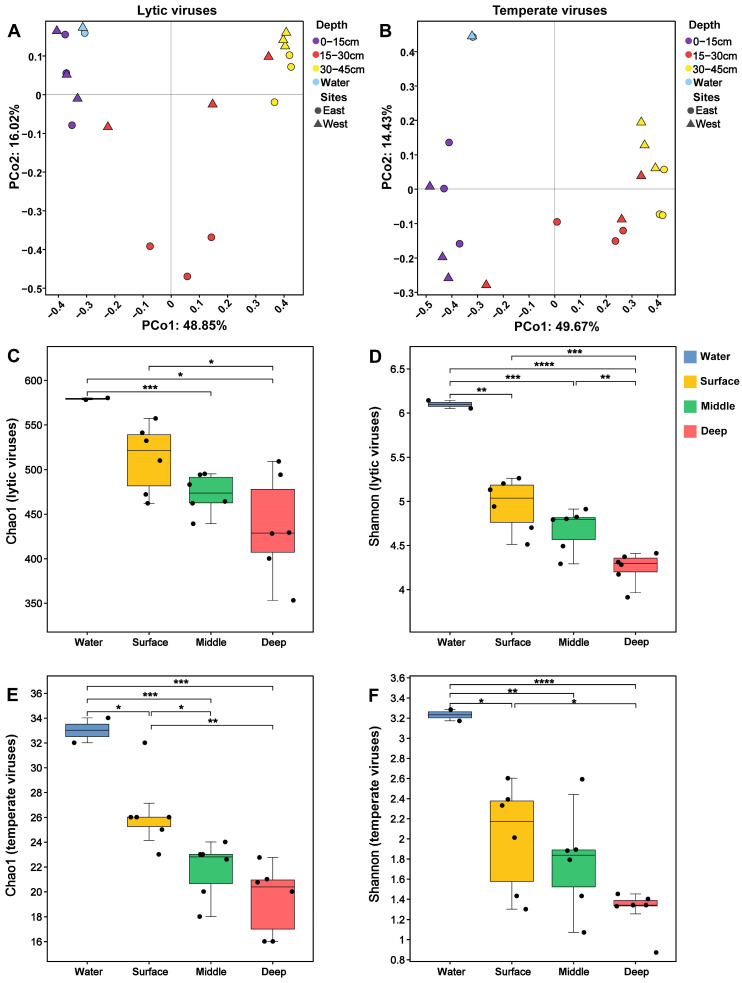
Viral community diversity. PCoA of lytic (**A**) and temperate (**B**) viral communities based on Bray–Curtis dissimilarity. Chao1 (**C**,**E**) and Shannon (**D**,**F**) indices of the different lifestyle viral community diversity. The number of asterisks denotes the statistical significance level (* *p* < 0.05, ** *p* < 0.01, *** *p* < 0.001, and **** *p* < 0.0001).

In order to explore the effects of factors such as different lake areas and depths on the differences in viral communities in Chaohu Lake, we performed beta diversity analysis. The results show that the community composition of lytic and temperate viruses does not differ significantly between the eastern and western central areas of the lake (PERMANOVA, *p* = 0.255, *p* = 0.226). Significant variances were observed in the viral community structures between sediment and lake water, encompassing both lytic and temperate viruses (PERMANOVA, *p* = 0.009, *p* = 0.008). All samples were categorized into three depth-based categories: surface (0–15 cm), middle (15–30 cm), and deep (30–45 cm). Analysis reveals that the composition of both lytic and temperate virus communities exhibits a substantial correlation with depth (*p* = 0.001, Figure 3A,B). Notably, minor discrepancies were detected between the lake water and surface sediments in terms of lytic virus composition, whereas more pronounced differences were observed between the lake water and surface sediments for temperate viruses.

The alpha diversity analysis reveals that lytic viruses exhibit higher diversity compared to temperate viruses (Figure 3C–F). The community diversity within the central region of the eastern lake was observed to be higher than that in the western lake’s center, though this variation was not statistically significant (Student’s *t* test, *p* > 0.05, Figure S5). There was a highly significant difference in Chao1 and Shannon indices between the Chaohu Lake water and sediments (Student’s *t* test, Figure 3C–F). Further examination of sediment depths indicates a reduction in the diversity of both lytic and temperate viruses with increased depth. This study also notes variations in virus community composition with depth, aligning with findings from previous research [43,50].

### 3.4. The Driving Factors of Virus Community Construction in Chaohu Lake

**Figure 4:**
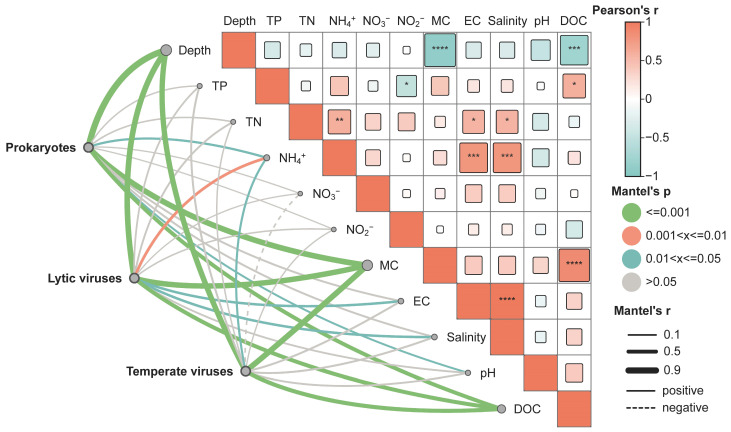
Environmental drivers of prokaryote, lytic, and temperate viral relative abundance. Line color represents Mantel’s *p* and line width represents Mantel’s r. The number of asterisks denotes the statistical significance level (* *p* < 0.05, ** *p* < 0.01, *** *p* < 0.001, and **** *p* < 0.0001). TP: total phosphorus; TN: total nitrogen; MC: moisture content; EC: electric conductivity; DOC: dissolved organic carbon.

While viruses are known to impact the structure and functional composition of microbial communities, the degree of this influence exhibits significant variability across different soil environments [52]. Through comprehensive analyses of viral abundance across various habitats within Chaohu Lake, along with consideration of environmental factors, it was observed that the community structure of temperate viruses exhibited a significant correlation with parameters such as ammonia nitrogen, moisture content (MC), depth, and dissolved organic carbon (DOC) levels (*p* < 0.05; Figure 4). Additionally, the structure of lytic virus communities showed significant correlations (*p* < 0.05) with conductivity, salinity, ammonia nitrogen, moisture content, depth, and DOC. Notably, moisture content, depth, and DOC levels are found to be highly significant (*p* ≤ 0.001) for prokaryote and viruses of both lifestyles, suggesting that these factors likely play a pivotal role in shaping viral community structure within Chaohu Lake.

### 3.5. Host Prediction of Chaohu Viruses

**Figure 5:**
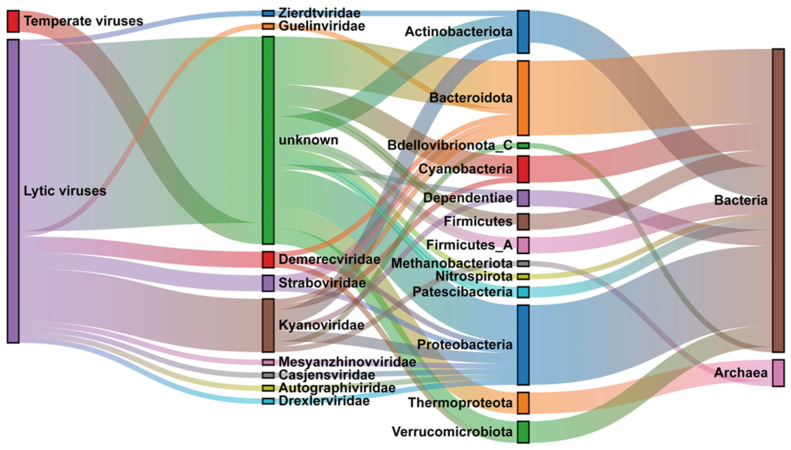
Predicted virus–host linkages. From left to right are viral lifestyle, viral taxonomy (family level), host taxonomy (phylum level), host taxonomy (domain level).

Understanding virus–host interactions contributes to our exploration of microbial community structure and ecological functions in freshwater lake sediments. In the quest to predict potential hosts for vOTUs in Chaohu Lake, our approach leverages MAGs derived from in situ assemblies, along with publicly accessible databases. Efforts to link vOTUs with their prospective hosts identified a total of 62 potential hosts, accounting for 9% of vOTUs. These hosts span 13 distinct phyla among bacteria and archaea (Figure 5; Table S7). Notably, only four temperate viruses among these exhibited predicted hosts, and the remaining predicted hosts were lytic viruses. Two or more prediction methods substantiated 26 virus–host pair associations. Approximately 97% of vOTUs were anticipated to infect specific hosts, while only two vOTUs demonstrated connections to hosts from distinct prokaryotic clades. This outcome aligns with the prevailing notion of a limited host range for the majority of viruses [18,53,54].

Our investigation revealed that among the vOTUs, five were linked to archaeal hosts, predominantly within the *Thermoproteota* lineage (*n* = 4), followed by a single instance in *Methanobacteriota*. In contrast, the prevalence of vOTUs was more pronounced in bacterial communities, with *Proteobacteria* leading (*n* = 15), succeeded by *Bacteroidota* (*n* = 14), *Actinobacteriota* (*n* = 8), *Cyanobacteria* (*n* = 5), and *Verrucomicrobiota* (*n* = 4). These taxa not only represent some of the most biomass-abundant but also metabolically vigorous bacterial lineages within the Chaohu ecosystem. This pattern is consistent with the ‘kill-the-winner’ hypothesis proposed by [2], which posits that the most dominant populations within bacterial communities are more susceptible to viral infections, thereby influencing microbial community dynamics and ecological balance.

### 3.6. Composition of AMGs for Different Lifestyle Viruses

Viruses induce cellular lysis, liberating significant quantities of dissolved organic matter, thereby stimulating microbial activity within aquatic ecosystems and modulating global biogeochemical cycling [55]. Beyond merely facilitating nutrient flux through predation, viruses possess the capability to influence the ecological dynamics of ecosystems via the horizontal transmission of ecologically pertinent genes and the expression of virus-encoded auxiliary metabolic genes (AMGs).

Utilizing VIBRANT and DRAM-v, we identified 177 and 141 putative AMGs, respectively, from Chaohu vOTUs. Following manual curation, lytic viruses were found to harbor 61 AMGs, while temperate viruses contained 14 AMGs (Tables S8 and S9). Notably, lytic viruses exhibited a higher species richness of AMGs compared to temperate viruses.

**Figure 6:**
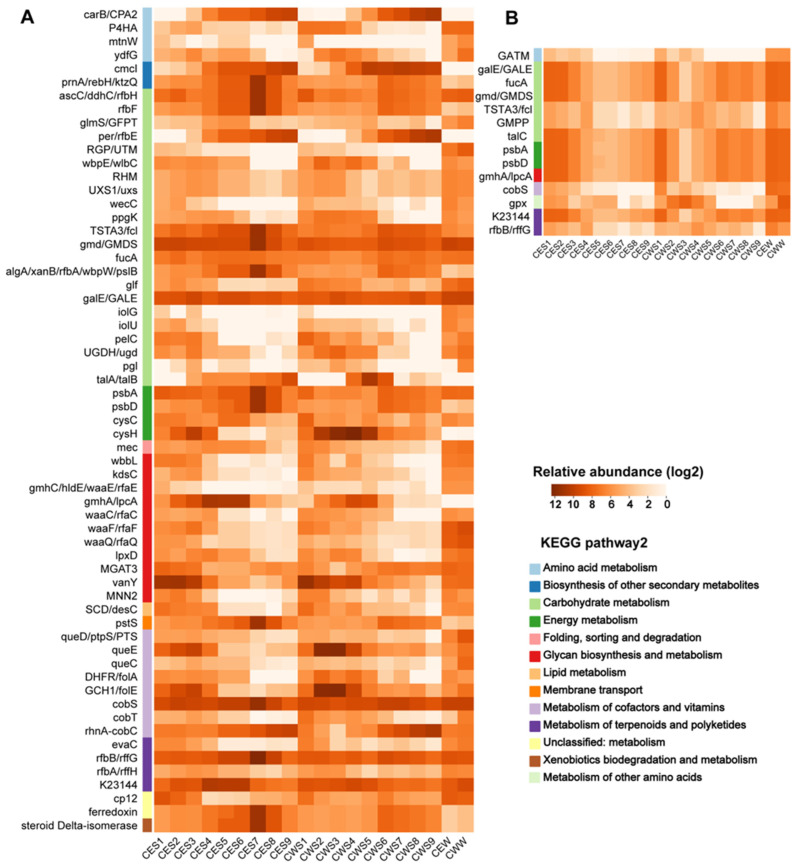
Function and abundance of AMGs. The relative abundances of AMGs of lytic (**A**) and temperate (**B**) viruses. The groups are labeled as follow: ‘CE’ and ‘CW’ means the central part of the eastern and western Chaohu Lake, respectively. The final letters ‘S’ and ‘W’ stand for sediment and lake water, respectively. Numbers from small to large represent layers from surface to deeper water.

Our investigation reveals that the majority of viruses isolated from Chaohu Lake carry class I AMGs. These virus-associated AMGs are implicated in the metabolic pathways of carbon and sulfur. Regarding temperate viruses, the AMGs are chiefly involved in the metabolism of carbohydrates, terpenoids, and polyketides. Conversely, lytic viruses predominantly engage in carbohydrate metabolism alongside glycan biosynthesis and metabolism (Figure 6).

## 4. Discussion

### 4.1. Linkages between the Chaohu Viruses and Other Ecosystems around the Globe

**Figure 7:**
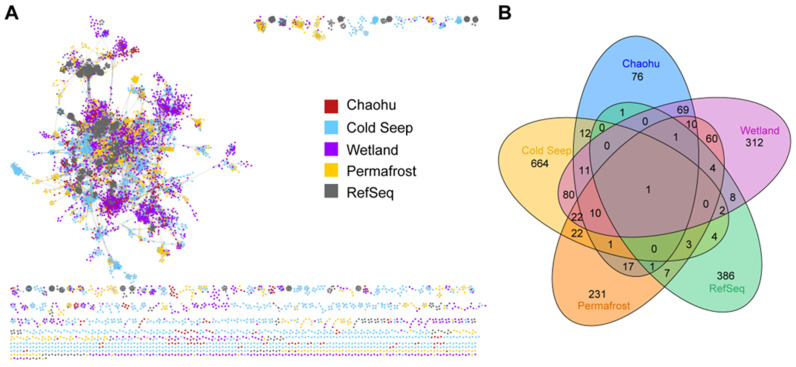
(**A**) Gene-sharing network of Chaohu Lake and other environmental virus sequences. (**B**) Venn diagram of shared viral clusters.

To explore the relationship of the Chaohu viruses with viral sequences from publicly available databases, we constructed gene-sharing networks using vConTACT2. A total of 373 Chaohu vOTUs (55.7%) could be clustered with the databases, and 297 vOTUs were categorized as singleton, outlier, and overlap (Figure 7A; Table S10). Specifically, 177 Chaohu vOTUs (47.5%) were clustered with wetland samples, a proportion higher than that observed in other environmental virus datasets (Table S11). Our analysis reveals a pronounced similarity between the viral communities in the Chaohu ecosystem and those found in wetland environments, indicating a closer phylogenetic relationship among viruses inhabiting analogous ecological niches. Notably, only a small fraction (1.3%) of the Chaohu vOTUs clustered with the Viral RefSeq database, consistent with previous studies [40,56]. More than half of the viruses are not classified, underscoring the presence of numerous novel and unidentified viruses within the Chaohu Lake ecosystem, warranting further exploration and investigation. The coexistence of 11 vOTUs in four ecosystems demonstrates that some of the viral communities may be widespread in different ecosystems across the globe and reveals the existence of phylogeographic connectivity between habitats (Figure 7B).

### 4.2. Driving Mechanism of Virus Community Construction in Chaohu Lake

Sediments represent the cumulative deposition of suspended materials within overlying water bodies, encapsulating the chronological evolution of ecological and environmental data. The two observation sites examined in this study are situated within the same aquatic system and share analogous sediment deposition timelines. Geographically, the viral community structures at equivalent depths from the eastern and western central regions of Chaohu Lake exhibits negligible disparity. This uniformity likely stems from the interconnected lake currents [57] and identical sedimentation timelines, fostering a higher homogeneity within the viral populations. Conversely, a vertical examination reveals distinct differences in viral community structures within Chaohu Lake, with the overlying water column and surface sediments displaying enhanced diversity and elevated community heterogeneity (Figure 3C–F). This suggests that viruses, alongside their respective hosts, may demonstrate heightened adaptability to environments rich in aquatic resources. It also underscores the influence of hydraulic disturbances and sedimentation timelines on the spatial distribution of these microbial communities. The abundance of organic material in the environments of overlying water bodies and surface sediments underpins the proliferation and sustenance of diverse viral entities, culminating in augmented viral activity within these contexts. Moreover, anthropogenic influences, such as urban wastewater and agricultural runoff, may introduce varied viral strains, thereby enriching the viral community diversity [58,59].

The correlation analysis between the viral and prokaryotic community structure and environmental factors shows that moisture content, depth, and DOC have a highly significant correlation with the prokaryotic and the two lifestyle viral community structures (*p* ≤ 0.001; Figure 4) in Chaohu Lake. This suggests that these environmental variables may play pivotal roles in shaping the viral communities within this aquatic ecosystem. DOC is a major carbon source for microorganisms in the water column, and moisture content and depth are closely related to important factors for microbial growth such as sediment DO and redox potential. We, therefore, hypothesize that environmental factors such as moisture content, depth, and DOC may be influencing the abundance and biodiversity of viruses in sediments by affecting the community structure and activity of virus hosts (bacteria and archaea). In addition, nutrient levels have been recognized as an important factor influencing the structure of viral communities in freshwater lakes [8]. Our findings underscore a significant positive association between ammonia nitrogen levels and both prokaryotic and lysogenic temperate viruses, underscoring the integral roles of viruses and their hosts within the nitrogen cycling processes of the Chaohu Lake ecosystem. It is noteworthy that pore water was not excluded from our samples, potentially contributing to the identification of some vOTUs directly originating from pore water [50,60].

Salinity is commonly acknowledged to influence viruses by regulating bacterial community structure and biomass, serving as a crucial environmental determinant shaping viral ecological distribution [7]. Our study, however, uncovered a notable association between salinity and the community structure of lytic viruses (*p* < 0.05), a correlation that was not evident in temperate viruses (Figure 4). In addition, we observed a substantial correlation between total phosphorus and the community structure of lytic viruses (*p* < 0.05), while such a relationship was absent in temperate viruses. This finding suggests a complex interplay warranting further comprehensive investigation.

### 4.3. Relationship between Viruses and Hosts

Viruses in Chaohu Lake may significantly influence the carbon and nitrogen biogeochemical cycling within their ecosystems. In our study, the predicted viral archaeal hosts are *Thermoproteota* and *Methanobacteriota*. *Thermoproteota* is widely distributed in a variety of ecosystems globally, including marine [61], soil [62], and freshwater [63], and plays an important role in nitrogen and carbon biogeochemical cycling [64]. Notably, our findings indicate that archaeal viruses infecting *Thermoproteota* exhibit significantly higher abundances compared to bacteriophages, with relative abundances reaching 6% in eastern Chaohu and escalating to 10% in western Chaohu. This trend is coupled with an increase in viral abundance correlating with sediment depth (Figure S6). On the other hand, *Methanobacteriota*, known for its methanogenic properties, suggests that archaeal viruses with the potential to infect methanogenic archaea could be prevalent in Chaohu Lake, potentially impacting methanogenic metabolism within this ecosystem. Despite the relatively lower abundance of archaea compared to bacteria in the Chaohu Lake ecosystem (Figure 1B), the prevalence of archaeal viruses was conspicuously higher in the middle and deep sediment layers (20–45 cm) (Table S7). This suggests a heightened susceptibility of archaea to viral infections in the Chaohu Lake ecosystem, a phenomenon that corroborates previous research indicating that archaeal infections by viruses occur at a rate of twice that of bacterial infections, despite the latter’s higher abundance [65]. Therefore, viruses may exert a critical regulatory influence on lake carbon cycling through the infection of archaea.

Our findings indicate that a predominant fraction of viruses targeting bacteria in Chaohu Lake’s waters are of the lytic type. The bacterial hosts, encompassing *Proteobacteria*, *Bacteroidota*, *Actinobacteriota*, *Cyanobacteria*, *Verrucomicrobiota*, and *Nitrospirota*, represent the most biomass-abundant and metabolically vigorous dominant bacterial phyla in both the aquatic and sedimentary environments of Chaohu Lake. Based on these observations, we propose that bacteriophages might exert a significant regulatory influence on both the structural succession and metabolic functions of Chaohu Lake’s microbial ecosystem. Given that Chaohu Lake is characterized by eutrophic conditions with a high prevalence of *Cyanobacteria*, bacteriophages potentially mitigate algal bloom outbreaks by infecting and lysing cyanobacterial cells. Furthermore, *Nitrospirota*, a critical microorganism in nitrogen metabolism [66], is influenced by viral infection, thereby modulating the nitrogen cycling within the lake’s aquatic system to a certain degree.

### 4.4. Differences in AMGs of Viruses with Different Lifestyles

AMGs typically encode key enzyme proteins that regulate crucial steps in host metabolism. They are considered to be the primary genetic sequences responsible for enabling viruses to manipulate host metabolism during infection, thereby promoting viral replication and eventual cell lysis [67]. Class I AMGs encode proteins associated with specific metabolic functions found within the Kyoto Encyclopedia of Genes and Genomes (KEGG) metabolic pathway. Class II AMGs are not represented in KEGG metabolic pathways [51].

Consequently, AMGs can exert indirect effects on host metabolic responses in addition to their direct involvement in metabolic processes. Our study finds that lytic viruses in Chaohu Lake may infect a wider range of host species and have a higher frequency of infections than temperate viruses, thus, possessing higher AMG diversity (Figure 6). This is consistent with previous reports [6,68].

Moreover, it has been demonstrated that the core photosystem II genes psbA and psbD are widespread among freshwater viruses, significantly contributing to the maintenance of active photosynthetic electron transport. This mechanism enhances the energy available for viral replication [69,70]. In conditions of elevated light intensity, the augmented expression of viral psbA genes facilitates an increase in translation efficiency and a reduction in the duration of the cleavage cycle [71]. Additionally, our analyses identified five vOTUs with the predicted host phylum being *Cyanobacteria*, all of which were categorized as lytic viruses (Figure 5; Table S7). This suggests that during lysogenic infections, viruses may express photosynthesis genes, sustaining photosynthetic activity in cyanobacteria throughout the viral lysogenic cycle and thereby ensuring adequate nutrient availability for viral propagation [72].

Typically, lytic viruses are known to hijack host metabolic pathways to expedite the biosynthesis of viral components [22,73]. In contrast, temperate viruses appear to bolster host survivability via auxiliary metabolic genes (AMGs) [21]. We found that temperate viruses from the lake water of Chaohu Lake carry the gene encoding glutathione peroxidase (gpx), which acts in cells to protect them from oxidative damage. This gene was absent in lytic viruses, indicating that AMGs in temperate viruses may enhance host resilience against harsh environmental conditions [74].

Viruses carry AMGs that affect host metabolism and, thus, the biogeochemical cycling of substances in lake. Lytic viruses carry the Calvin cycle repressor gene, cp12, which can redirect energy flow to metabolic processes in the host, directing carbon flux in host cells from the Calvin cycle to the pentose phosphate pathway (PPP) [72]. The PPP produces NADPH, pentose, and ribulose-5-phosphate, which provides nucleotides for virus replication. This process leads to an increase in viral lysis, which, in turn, enhances its fitness [75]. The prevalence of AMGs associated with carbon metabolism in Chaohu viruses implies that the virus may play a key role in the microbe-mediated carbon cycling in Chaohu [76]. AMGs associated with sulfur metabolic pathways, such as those encoding phosphoadenosine phosphosulfate reductase (cysH) and adenylylsulfate kinase (cysC), are also present in lytic viruses [18], and they are important components of the sulfur cycle in the Chaohu Lake ecosystem. These findings highlight the extensive involvement of viruses in metabolic pathways, underlining their significance in the ecosystem’s biogeochemical cycling.

Acknowledging that certain predicted AMGs may not represent genuine AMGs is critical. Given their likely derivation from cellular entities, completely ruling out false-positive predictions concerning viral overlapping populations remains challenging. Therefore, further research, including genomic context assessment and functional analyses of these putative AMGs [77], is essential for a more detailed understanding of viral impacts on the Chaohu Lake ecosystem.

## 5. Conclusions

In summary, in this paper, we systematically investigated the abundance, composition, diversity, host relationship, and potential functional roles of viruses in Chaohu Lake. We discovered distinct compositional variations across viral communities associated with different lifestyles, with lytic viruses exhibiting significantly greater diversity than their temperate counterparts. Factors such as depth, moisture content, DOC, prokaryotic community structure, and nutrient conditions may be the main drivers of different lifestyle viral community structure distribution and succession. Lytic viruses, capable of infecting a broader spectrum of hosts and possessing a richer array of auxiliary metabolic genes (AMGs), tend to replicate autonomously, whereas temperate viruses potentially augment host survival via AMG mediation. By infecting the host and regulating host metabolic processes, viruses in Chaohu Lake are posited to critically influence carbon, nitrogen, and sulfur metabolism. These insights significantly advance our understanding of ecological succession patterns among viruses of varying lifestyles in freshwater lake sediments and their environmental implications.

## Data Availability

The data generated in the current study are publicly available. The amplicon sequence files have been deposited in the NCBI Sequence Read Archive database (SRA) BioProject PRJNA1077139. The metagenomic sequence files are available at the NCBI SRA BioProject PRJNA838605.

## Supplementary Materials

The following supporting information can be downloaded at: https://www.mdpi.com/article/10.3390/v16040590/s1, Figure S1: Sampling sites in Chaohu Lake; Figure S2: Flowchart of bioinformatic processing. Figure S3: Genome quality of vOTUs from Chaohu Lake; Figure S4: The common and unique vOTUs between eastern and western Chaohu Lake water. Figure S5: Chao1 index (A) and Shannon index (B) for lytic viruses, Chao1 index (C), and Shannon index (D) for temperate viruses; Figure S6: Relative abundance of viruses whose host is Thermoproteota; Table S1: Summary of clean data statistics of metagenomic sequencing and physicochemical data; Table S2: Assembly qualities of the contigs; Table S3: Physicochemical data of the Chaohu Lake samples; Table S4: Detailed information of the MAGs; Table S5: Detailed information of the vOTUs identified in samples; Table S6: Relative abundance (expressed as RPKM values) table for 670 vOTUs in each sample; Table S7: Detailed information of virus–host linkages prediction; Table S8: Annotation and abundance of the lytic viral AMGs; Table S9: Annotation and abundance of the temperate viral AMGs; Table S10: Clustered vOTUs among Chaohu Lake, other environmental virus sequences, and viral RefSeq based on vConTACT2; Table S11: Occurrence of viral clusters among Chaohu Lake, other environmental virus sequences, and viral RefSeq based on vConTACT2.
