# Supplementary material for: Community Structure, Drivers, and Potential Functions of Different Lifestyle Viruses in Chaohu Lake
Source: Viruses. 2024 Apr 11;16(4):590. doi: 10.3390/v16040590 (PMC11053968; doi:10.3390/v16040590)
Supplement: Supplementary file 1 [file viruses-16-00590-s001.zip › Supplementary materials.pdf]

Community Structure, Drivers, and Potential Functions of Different Lifestyle Viruses in Chaohu Lake

Yu Zheng [1, 2], Zihao Gao [1, 2], Shuai Wu [1, 22] and Aidong Ruan [1, 3, *]

[1] The National Key Laboratory of Water Disaster Prevention, Hohai University, Nanjing 210024, China.

[2] College of Hydrology and Water Resources, Hohai University, Nanjing 210098, China.

[3] College of Geography and Remote Sensing, Hohai University, Nanjing 210098, China.

[*]Corresponding Author: Aidong Ruan

E-mail address: adruan@hhu.edu.cn

Introduction to the Chaohu Lake

Chaohu Lake is located in the middle and lower reaches of the Yangtze River system, with a watershed population of over 10 million. Its western water is more seriously polluted, with nitrogen and phosphorus levels and heavy metal concentrations significantly higher than those in the eastern waters. Chaohu Lake is an important pivotal water body connecting the Huaihe River basin and the Yangtze River basin, and its watershed area is one of the most densely populated and economically active regions in China. Virus abundance, distribution and diversity not only record the ecological evolution of the upstream area, but also have an important impact on the health of the population and the ecological environment in the neighborhood and the middle and lower reaches of the Yangtze River.

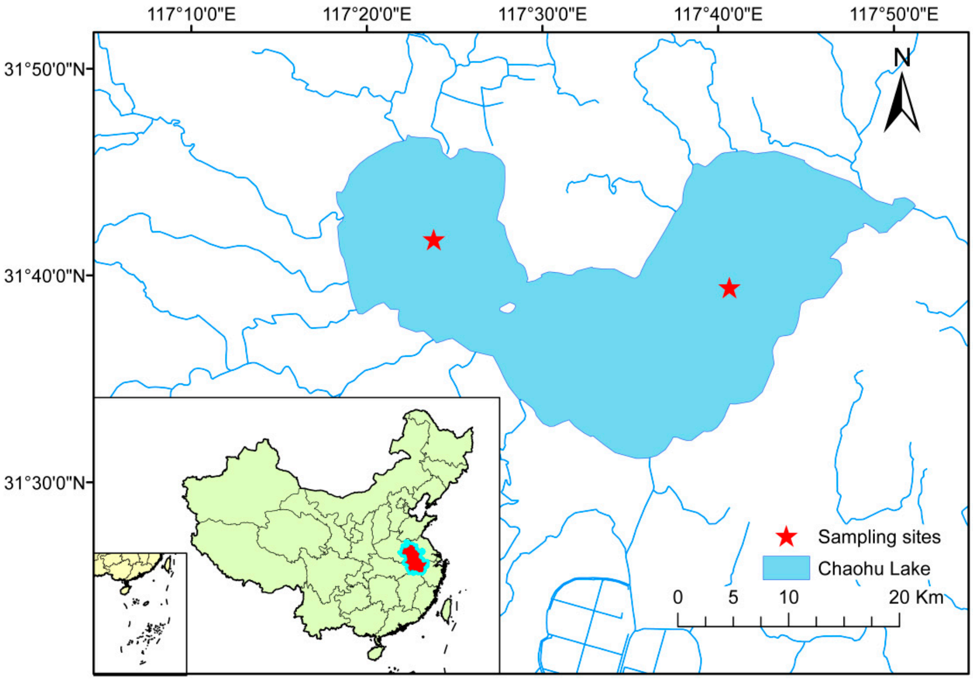

Figure S1. Sampling sites in Chaohu Lake.

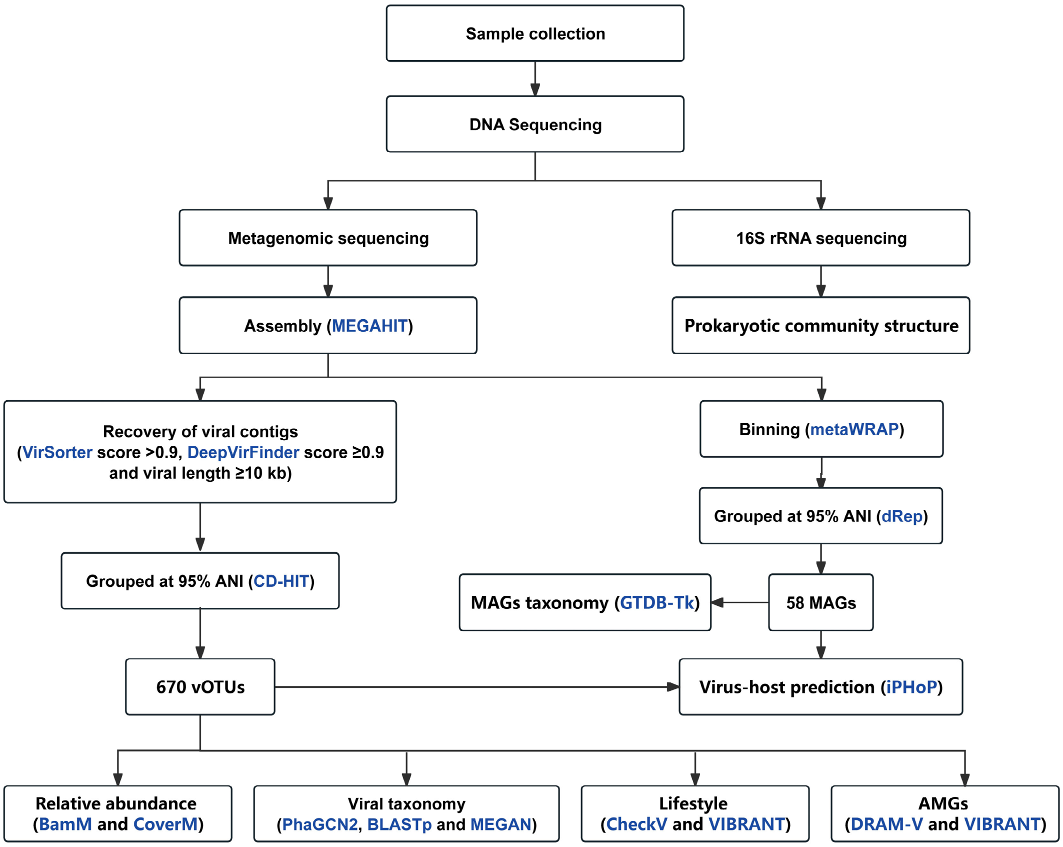

Figure S2. Flowchart of bioinformatic processing. Software is represented in blue.

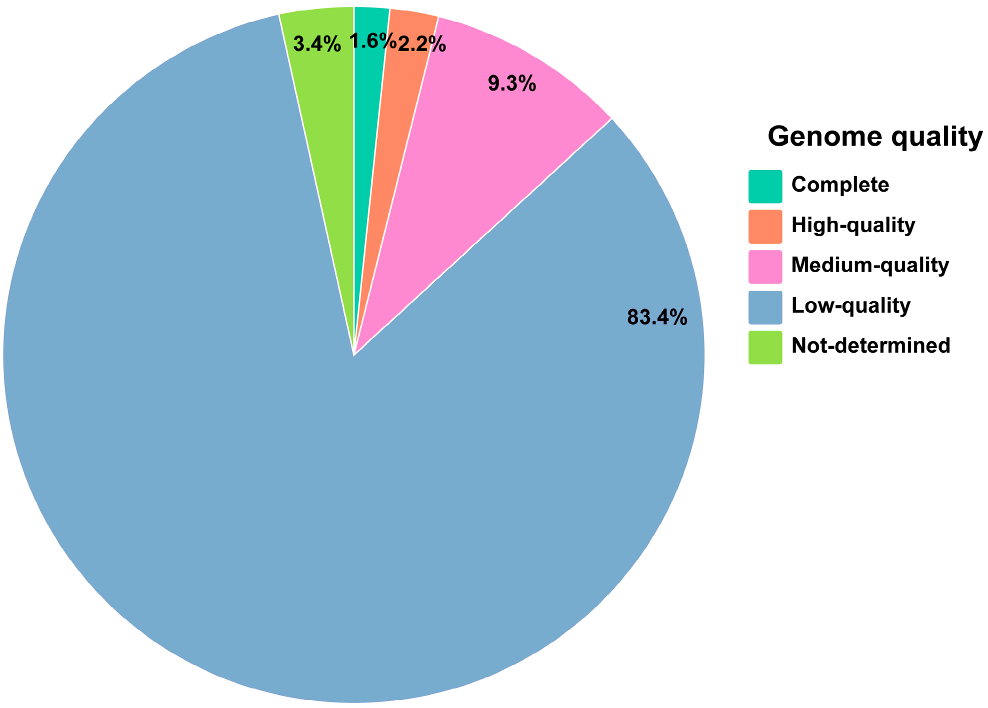

Figure S3. Genome quality of vOTUs from Chaohu Lake.

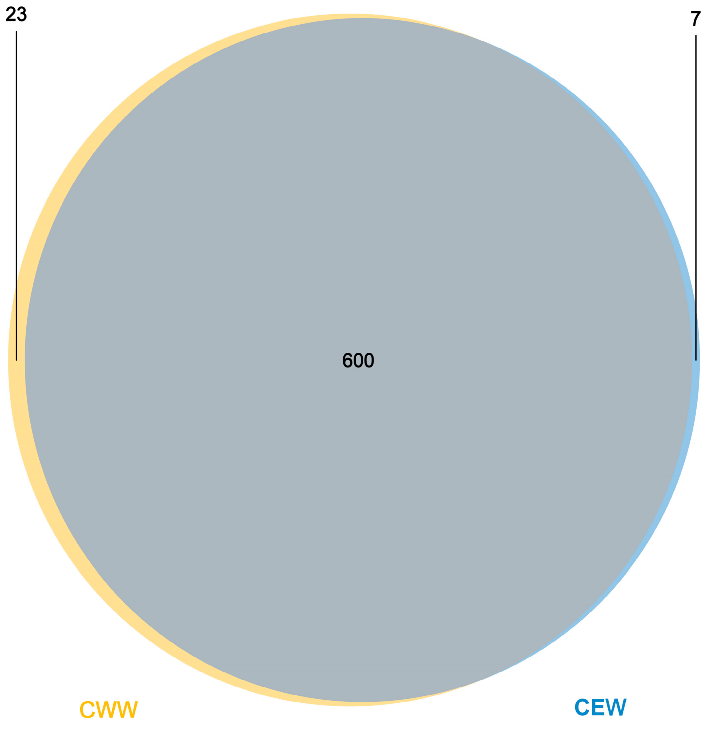

Figure S4. The common and unique vOTUs between eastern and western Chaohu Lake water.

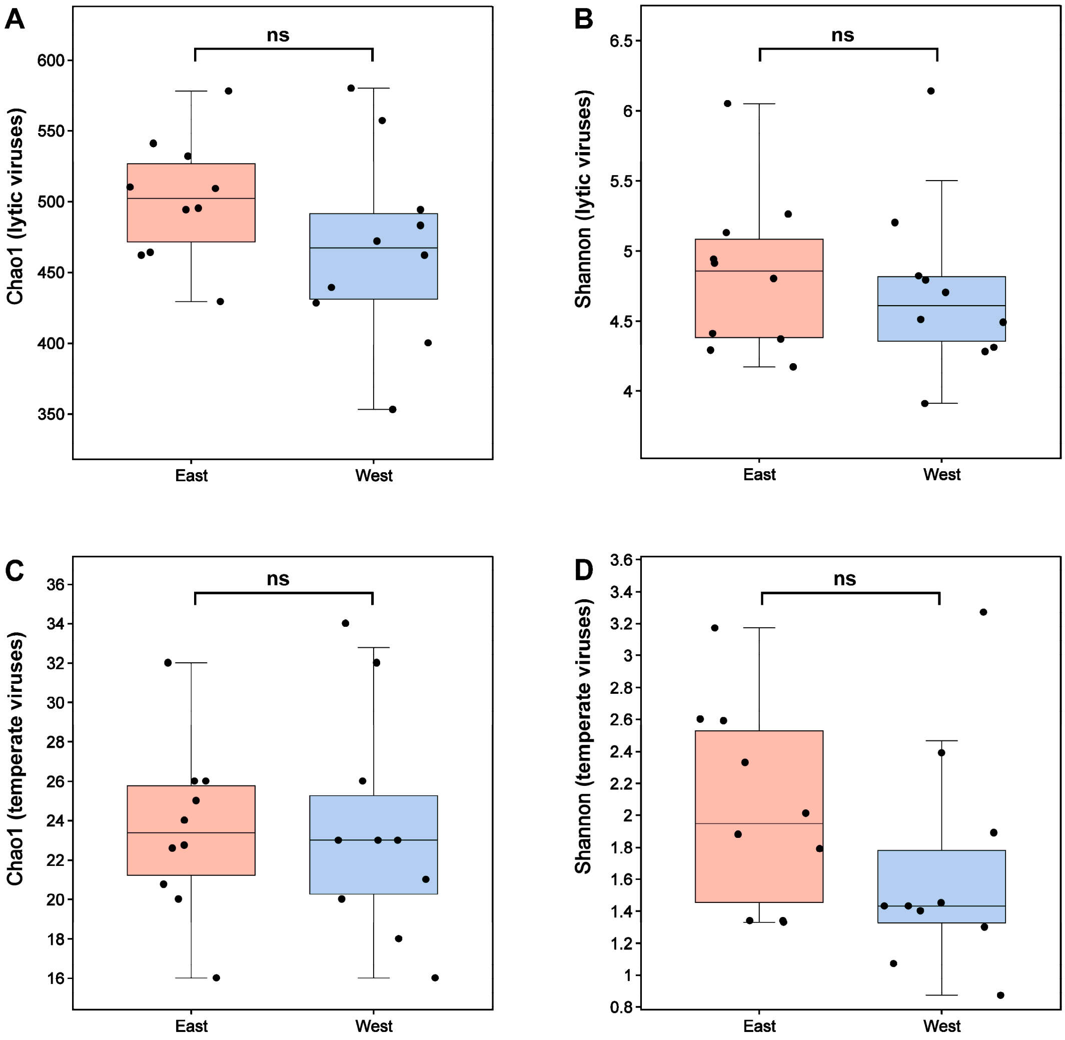

Figure S5. Chao1 index (A) and Shannon index (B) for lytic viruses, Chao1 index (C) and Shannon index (D) for temperate viruses.

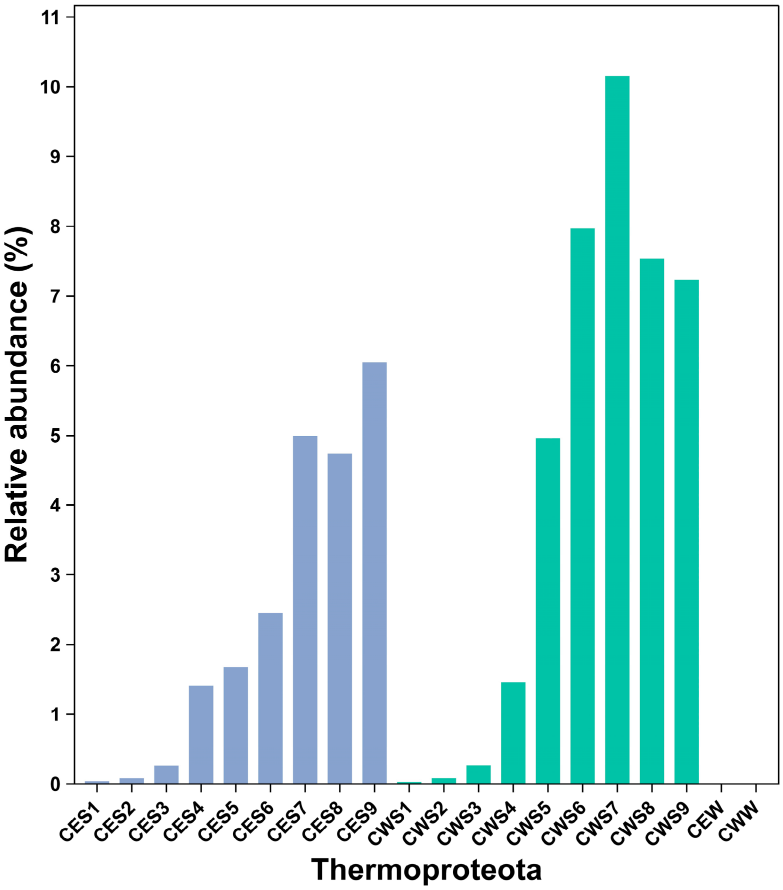

Figure S6. Relative abundance of viruses whose host is *Thermoproteota*.
